# Ubiquitin specific peptidase 11 as a novel therapeutic target for cancer management

**DOI:** 10.1038/s41420-022-01083-5

**Published:** 2022-06-17

**Authors:** Yihao Liao, Diansheng Zhou, Pu Wang, Mengyue Yang, Ning Jiang

**Affiliations:** 1 Tianjin Institute of Urology, The Second Hospital of Tianjin Medical University, Tianjin, 300211 China; 2 Department of Urology, The Second Hospital of Tianjin Medical University, Tianjin, 300211 China; 3 Department of Cardiology, The Second Affiliated Hospital of Harbin Medical University, Harbin, Heilongjiang province 150000 China

**Keywords:** Tumour biomarkers, Targeted therapies

## Abstract

Ubiquitination is a critical biological process in post-translational modification of proteins and involves multiple signaling pathways in protein metabolism, apoptosis, DNA damage, cell-cycle progression, and cancer development. Deubiquitinase, a specific enzyme that regulates the ubiquitination process, is also thought to be closely associated with the development and progression of various cancers. In this article, we systematically review the emerging role of the deubiquitinase ubiquitin-specific peptidase 11 (USP11) in many cancer-related pathways. The results show that USP11 promotes or inhibits the progression and chemoresistance of different cancers, including colorectal, breast, ovarian, and hepatocellular carcinomas, via deubiquitinating several critical proteins of cancer-related pathways. We initially summarize the role of USP11 in different cancers and further discuss the possibility of USP11 as a therapeutic strategy.

## Facts

1. USP11 has exerted critical role in multiple cancer occurrence and progression in various studies.
2. The drugs could be designed based on USP11 structure and function for clinical application.
3. We preliminary summarized the role of USP11 in different cancers and further discussed the possibility of targeting USP11 as therapeutic strategies.

## Open questions

1. Whether USP11 has other functions except deubiquitinase?
2. Whether USP11 has other unusual deubiquitin manner liking deubiquitinase OTUB1 apart from the classical manner?
3. USP11-specific inhibitor need to further research for clinical application especially in these advanced cancer patients.

## Background

**Fig. 1:**
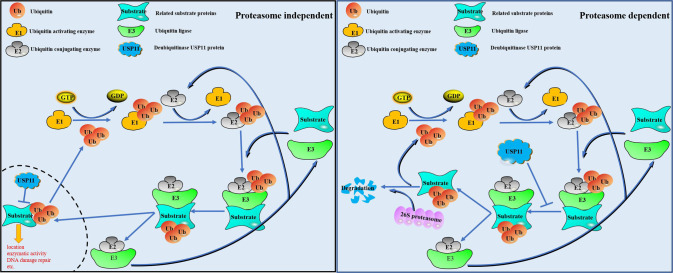
The flow chart of ubiquitination and the mechanism of ubiquitination inhibition by USP11. Left panel: proteasome-independent manner, ubiquitin is activated by E1 and attaches to E1, which is further transferred to E2 and forms a new complex. The entire complex then binds to the E3 substrate complex to form a complete ubiquitination complex, and E3 eventually transfers the ubiquitin molecule to the substrate protein. USP11 further cleavages ubiquitin chain from substrate protein (such as lys63- and lys6-linked ubiquitin chain), eventually contributed to the change of substrate protein function and location etc. not degradation; right panel: proteasome-dependent manner, ubiquitin is activated by E1 and attaches to E1, which is further transferred to E2 and forms a new complex. The entire complex then binds to the E3 substrate complex to form a complete ubiquitination complex, and E3 eventually transfers the ubiquitin molecule to the substrate protein, where the substrate bound ubiquitin further recruit proteases for substrate degradation. USP11 significantly inhibits substrate protein degradation (usually lys48-linked ubiquitin chain)).

Post-translational and post-transcriptional modifications are the most important and prevalent modifications in a variety of signaling pathways that play enormous roles in physiological and pathological processes [1, 2]. post-transcription modifications mainly include acetylation and methylation, which regulate the transcription of various factors and further trigger normal or abnormal activation of critical factors. Phosphorylation and ubiquitination are considered as the most common post-translational modifications, and they are both instantaneous processes that exert precise regulatory effects. Phosphorylation frequently occurs in proteins central to these important signaling pathways, leading to the activation of associated proteins and further corresponding functions. While ubiquitination is one of the most critical protein degradation methods, which guarantees that activated proteins are degraded by proteases timely and abnormal protein products are removed rapidly [3, 4]. Ubiquitination, including E1 ubiquitin-activating enzymes, E2 ubiquitin-conjugating enzymes, and E3 ubiquitin ligases, usually refers to the whole process in which ubiquitin molecules are activated and transferred to the substrate proteins, which in turn recruits relevant proteases to degrade the substrate protein. First, ubiquitin is activated by E1 and attaches to E1, which further transfers to E2 and forms a new complex. Then, the whole complex binds to E3 substrate complex to form a complete ubiquitinated complex, and E3 finally transfers the ubiquitin molecule to the substrate protein, and the substrate binds ubiquitin to further recruit proteases for substrate degradation. Finally, the substrate protein is degraded, and the remaining ubiquitin molecules and corresponding ubiquitinases are separated for the subsequent ubiquitination process [5] (Fig. 1). Ubiquitination has been reported to be involved in numerous physiological and pathological processes, especially in the occurrence and development of various cancers [3], which mainly depends on the ubiquitination process of central carcinogenic or oncogenic genes. Among these ubiquitinases, E3 ubiquitin ligase is the most critical element because it can directly interact with the substrate protein and determine the specificity of the substrate protein [6]. In turn, the dynamic ubiquitination process is also precisely regulated by deubiquitylating enzymes (DUBs), which have been identified to regulate the progression of various cancers and other diseases via removing ubiquitin from the substrate or restraining the conversion of ubiquitin from E2 to E3. Currently, these are ~100 DUBs from 7 different families [7], containing the Ubiquitin-Specific Proteases (USPs) superfamily, the Machado-Joseph Domain-containing Proteases (MJDs) superfamily, the Ovarian Tumor proteases (OTUs) superfamily, the JAMM/MPN Domain-associated Zinc-depend Metalloproteases (JAMMs) superfamily, the Ubiquitin C-terminal Hydrolases (UCHs) superfamily, the Zinc Finger Containing Ubiquitin Peptidase 1 (ZUP1) superfamily and the Ubiquitin Containing Proteases (MINDYs) superfamily [8, 9]. The USPs family is the largest and most important DUB family, and many of these elements are involved in the occurrence and development of multiple cancers, such as USP1, USP4, USP7, USP22, and USP28, which promote or inhibit the development and progression of colorectal, breast, and hepatocellular carcinomas via deubiquitining the hub genes of cancer-related signaling pathways [10, 11]. The roles of various USPs in the occurrence and development of multiple cancer-related signaling pathways have also been reviewed and summarized. Recently, USP11 is also a member of the USPs family that is emerging as central to the regulation of various cancer progression. In this paper, we systematically summarize the role of USP11 in various cancer-related signaling pathways and focus on the targeting of USP11 as a potential therapeutic strategy for a variety of cancers.

## The structure and function of USP11

**Fig. 2:**
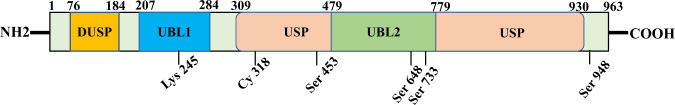
The structure of USP11. The schematic diagram of amino acid sequence, domain, and critical amino acid sites.

Protein ubiquitination controls many intracellular processes, including cell cycle progression, transcriptional activation, and signal transduction. Deubiquitinating enzymes are cysteine proteases that specifically cleave ubiquitin from ubiquitin-conjugated protein substrates [12–14]. USP11 encodes a deubiquitinating enzyme that is located in a gene cluster on chromosome Xp11. 23,963 amino acids and ~109,817 Da [15]. There are two critical domains involved in the catalytic and binding activities of deubiquitinating enzyme, DUSP and USP domains [16, 17]. The cysteine 318 site is the core amino acid, involved in catalytic activity, and the mutation or deletion of cysteine 318 site results in the loss of deubiquitinating function [16, 18, 19] (Fig. 2). As a pivotal component of USPs family, USP11 can remove conjugated ubiquitin or polyubiquitin chains from substrate proteins and exhibit specificity for ubiquitin chains. Ubiquitin chain cleavage assays with all eight linkages revealed USP11 preference for Lys63-, Lys6-, Lys33-, and Lys11-linked chains over Lys27-, Lys29-, and Lys48-linked and linear chains, which is consistent with USP11’s function in DNA repair pathways that is regulated by the protease domain. The results further indicated its specificity whereby USP11 domains outside the catalytic core domain serve as protein interaction or trafficking modules rather than a direct regulatory function of the proteolytic activity. This highlights the diversity of USPs in substrate recognition and regulation of ubiquitin deconjugation [15, 16]. The ubiquitin chains preferentially cleaved by USP11 tend to present different conformations. Lys63-linked chains have tendency to adopt extended conformations, whereas Lys6-linked chains present relatively compact conformations. Both Lys63-linked and linear chains generally adopt relatively open conformations. A high degree of flexibility and the linkage surrounding residues, such as the relatively bulky methionine in the linear chain, might be responsible for the differences. Moreover, Lys63- and Lys6-linked ubiquitin chains have been associated with pathways that USP11 has been implicated in DNA damage repair and inflammation, but the functional significance of this in vivo remains to unclear. Preferential cleavage of ubiquitin chains in USP11 is not comparable to the specificity of other deubiquitinating enzymes (such as OTUB1). USP11 also is identified to cleavage some extent with all chains except linear diubiquitin chain (linear diubiquitin chain displayed little cleavage activity). All USP11 constructs consistently show less activity toward Lys27-, Lys29-, and Lys48-linked and linear chains and as such a clear preference for some chains over others. Current understanding supports the regulation and function of USP11 in DNA damage repair pathways and viral infection. Lys48-linked ubiquitin chain is common associated with proteasome-dependent protein degradation, while lys63-linked ubiquitin chain is related to multiple proteasome-independent processes such as DNA damage response, protein interaction, and enzymatic activity etc. USP11 has been identified to prefer to lys63-inked ubiquitin chain and associated with DNA damage response and inflammation signaling. Although USP11 has relatively less cleavage activity to lys48-linked ubiquitin chain, it is also identified to regulate the occurrence and progression of several cancers via stabilizing protein expression, which depends on lys48-linked cleavage (Fig. 1). The ubiquitination of many critical proteins associated with signaling pathways will contribute to the occurrence or progression of different diseases, and USP11 typically stabilizes these factors via inhibiting the degradation process and further promoting or inhibiting the disease state. Previous studies have demonstrated that USP11 not only regulates DNA damage repair [20–24] but also immune cell differentiation via NF-κB [25, 26] and tumor necrosis factor (TNF) signaling pathways [27]. Recently, an increasing number of researchers have found that USP11 is involved in the occurrence and development of multiple cancers, such as colorectal, breast, hepatocellular, and ovarian cancers, implying that USP11 is a potential therapeutic target for a variety of cancers.

In this review, we summarize the current existence of USP11 in pathophysiological conditions (Table 1). As a typical deubiquitinating enzyme, USP11 promotes or inhibits the occurrence and progression of different diseases by deubiquitinating different proteins, depending on its deubiquitinating enzyme catalytic activity. Based on the current research, we found that USP11 can remove K-48 and K-63 ubiquitin connection types from substrate proteins. For example, Xiaolin Zhu et al. [28] identified that USP11 promotes ovarian cancer chemoresistance by removing the K48 ubiquitin chain of BIP. Mazin A Al-Salihi et al. [29] found that USP11 augmented TGFβ signaling by removing the K48 ubiquitin chain of ALK5. Moreover, Heeyoung Yang et al. [30] found that USP11 regulates liver disease status by removing the K63 ubiquitin chain of KLF4; and Dan Wang et al. [31] identified that USP11 drives Peg10 gene expression and activates lung epithelial cells by removing the K63 ubiquitin chain of phosphorylated E2F1. Numerous studies have proved that USP11 regulates the occurrence and development of different diseases through its deubiquitination function, and the specific type of ubiquitin linkage needs to be further investigated. Notably, USP11 plays a key role in DNA damage repair depending on its catalytic activity, for example, Xia Ting et al. [20] found that USP11 acts as a histone deubiquitinase in chromatin reorganization during DNA repair, Palak Shah et al. [21] found that USP11 regulates UV-induced DNA damage repair by deubiquitinating XPC, and Tanggang Deng et al. [32] identified that the deubiquitylation and stabilization of p21 by USP11 is essential for cell cycle progression and DNA damage responses. The above results suggest that USP11 plays an extremely important role in DNA damage repair, and the specific type of ubiquitin linkage needs to be further investigated. PML [33], SOX11 [18], VCP [34], and other proteins were also proved to be deubiquitinated by USP11 with uncertain linking type, which further contributed or restrained the progression of different diseases. Meanwhile, previous researchers have found that USP11 regulates several factors and related signaling pathway, such as RhoA [35] and Erα [36], but the specific manner remains unclear. Intriguingly, the activity of USP11 could be phosphorylated by FASN-induced PI3K-S6 kinase signaling, and phosphorylated USP11 further enhances its interaction with eIF4B and thereby promoting oncogenic translation [37], implying that the activity of deubiquitinase USP11 is also activated by the kinase and enriches the complex regulatory network of USP11. Weitong Zhang et al. [38] found that Circ_DOCK1 interference suppressed USP11 by increasing miR-132-3p, thereby inhibiting cell growth and metastasis and increasing apoptosis in colorectal cancer, which further identified the novel interaction between miRNA and USP11. The above results demonstrate that USP11, a typical deubiquitinase, regulates the state and stability of multiple proteins via several manners. Researchers can design more precise strategies to inhibit the effects of USP11 based on specific activated and functional patterns.

**Table 1:** The different manners of USP11 in pathophysiological conditions.

Manner		Interacting factors	Results	References
Deubiquitination depending on the USP domain of USP11	K-48 ubiquitin chain	BIP	USP11 promotes ovarian cancer chemoresistance by removing the K48 ubiquitin chain of BIP.	[28]
		ALK5	USP11 augments TGFβ signaling by removing the K48 ubiquitin chain of ALK5.	[29]
	K-63 ubiquitin chain	KLF4	USP11 regulates the status of liver diseases by removing K63 ubiquitin chain of KLF4.	[30]
		E2F1	USP11 drives Peg10 gene expression and activates lung epithelial cells by removing the K63 ubiquitin chain of phosphorylated E2F1.	[31]
	Uncertain ubiquitin linking type	H2AK119 H2BK120	USP11 acts as a histone deubiquitinase functioning in chromatin reorganization during DNA repair.	[20]
		XPC	Regulation of XPC deubiquitination by USP11 in repair of UV-induced DNA damage.	[21]
		P21	Deubiquitylation and stabilization of p21 by USP11 are critical for cell-cycle progression and DNA damage responses.	[32]
		PML, SOX11, VCP, etc.	USP11 promotes or inhibits the occurrence and progression of different diseases depending on its deubiquitination enzyme activity.	[18, 33, 34]
Uncertain manner		RhoA	USP11 promotes the chemotherapy resistance of gastric cancer by promoting RhoA and Ras mediating pathway.	[35]
		ERα	USP11 promotes the progression of breast cancer via regulating the activity of ERα.	[36]
Kinase modulating activity		P70-S6K; EIF4B	FASN-induced PI3K-S6 kinase signaling phosphorylates USP11 enhancing its interaction with eIF4B and thereby promoting oncogenic translation.	[37]
MiRNA binding USP11		Circ_DOCK1 miR-132-3p	Circ_DOCK1 interference suppressed cell growth and metastasis, and increased apoptosis of colorectal cancer via decreasing USP11 by increasing miR-132-3p.	[38]

## The regulation of USP11

The ubiquitinating and deubiquitinating processes are highly dynamic, transient and precisely regulated by multiple factors and related signaling pathways [39, 40]. Once signaling or function is complete, multiple core proteins will be ubiquitinated timely, thus ensuring complete signaling and avoiding overreaction [4, 41]. The disruption of USP11 leads to abnormal prolonged activation of the inactivation of certain factors, which in turn affects the occurrence or alteration of diseases. USP11 regulates ubiquitination, which is also regulated by different factors and manners, with acetylation [42] and phosphorylation [37] being the most common modifications. Chunaram Choudhary et al. found that USP11 might be acetylated at the Lys 245 site via extensive acetylation and mass spectrometry analysis [43], the specific experimental basis of which needs further validation. Furthermore, USP11 was also identified to be phosphorylated at Ser 648, 733, and 948 sites based on large-scale phosphorylation analysis and mass spectrometry [44–47], and the related kinases will be identified soon. Bandish Kapadia et al. [37] found that S6 kinase mediates the phosphorylation of USP11 at Ser453 site and further regulates the deubiquitinase activity. USP11 deubiquitinates and stabilizes the translation initiation factor EIF4B to promote EIF4B-dependent oncogenic translation, thus phosphorylating USP11 enhances the stability and abundance of EIF4B, which ultimately facilitates the occurrence of lymphoma. In addition to acetylation and phosphorylation, Weitong Zhang et al. [38] found that in colorectal cancer, the transcription process of USP11 was repressed by MiR-132-3p, while was further is restrained by Circ-DOCK1. USP11 is not only a ubiquitinating enzyme, but it has also been identified to interact with another common USPs (USP7), and the complex relationship between USP11 and USP7 may further strengthen this multiple effect and activity [24, 48, 49]. The above results demonstrate that the deubiquitinase USP11 depends on its deubiquitination function to regulate the occurrence and development of various physiological and pathological processes, but is also precisely regulated by multiple factors and modifications (Fig. 2; Table 2). All factors combine and form a complicated USP11-related regulatory network.

**Table 2:** The modification sites of USP11.

Sites	Modification manners	Reference
Lys245	Acetylation	[43]
Ser453	Phosphorylation	[37]
Ser648	Phosphorylation	[44, 45]
Ser733	Phosphorylation	Non (via forecast)
Ser948	Phosphorylation	[46, 47]

## USP11 in cancers

**Fig. 3:**
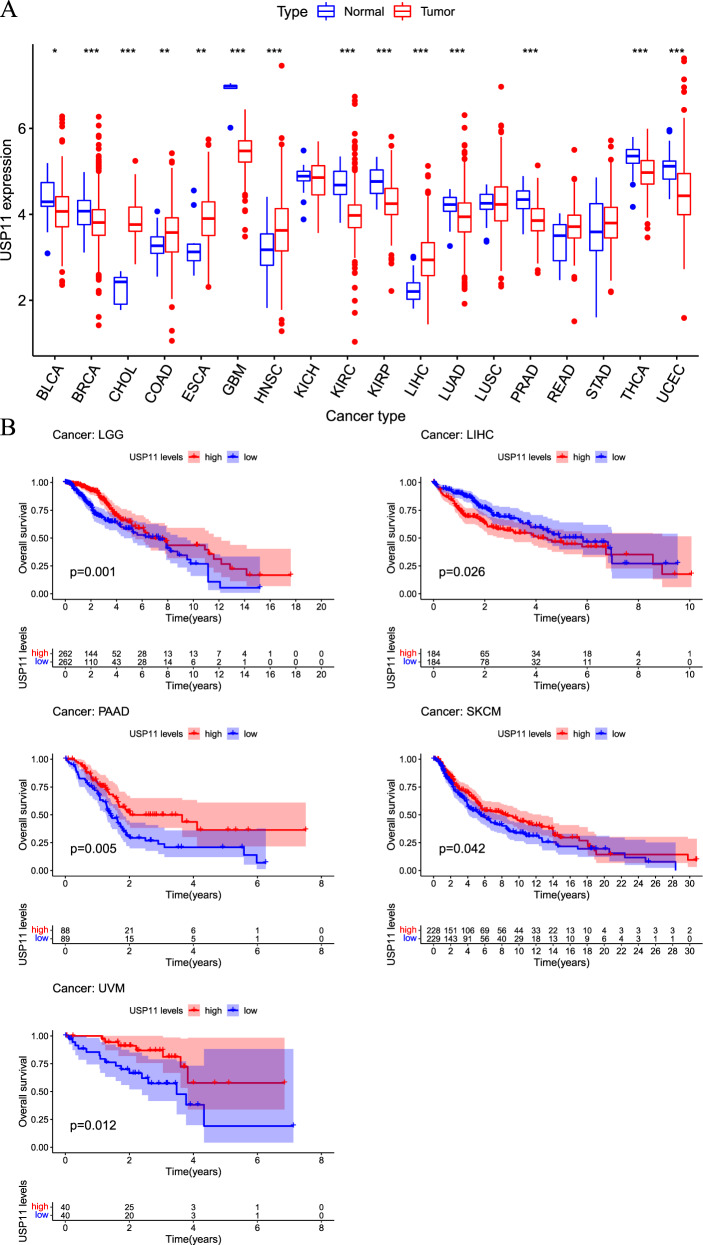
USP11 expression in pan-cancer and paired para-cancer tissue and Kaplan–Meier analysis. **A** The expression of USP11 in pan-cancer and paired para-cancer tissues based on bioinformatics analysis. **B** Kaplan–Meier analysis between USP11 expression and low-grade glioma (LGG); liver hepatocellular carcinoma (LIHC), pancreatic cancer (PAAD), skin cutaneous melanoma (SKCM), and uveal melanoma (UVM).

In previous studies, DUBs have been identified to be involved in multiple cancer-related signaling pathways and cancer prognosis, part of which have been applied to clinically targeted therapies. For instance, OTU Domain-Containing Ubiquitin Aldehyde-Binding Protein 1 (OTUB1), a member of OTUs family, has been identified as a typical cancer-related gene and regulates the occurrence and progression of various cancers including colorectal cancer [50, 51], prostate cancer [52, 53], breast cancer [54], and lung cancer [55, 56]. Silencing OTUB1 can significantly inhibit cancer status and metastasis, and targeting OTUB1 would be an excellent target for numerous cancer patients. Besides OTUB1, other DUBs, such as USPs family (USP1, USP7, USP8, USP15, USP22) [57–67], OTUs family (OTUD7B, OTUD6B, A20, and OTUB2) [68–75] and other DUBs have been identified to regulate the progression of various cancers and applied for clinical cancer therapy. Recently, USP11 has rapidly emerged as an important cancer-related regulator, and these studies have shown that USP11 relies on its deubiquitinating enzyme catalytic activity to regulate the occurrence and progression of a variety of cancers. The dysregulation of USP11 is related to multiple diseases and pathological processes, the expression of USP11 in multiple cancer and para-cancer tissue are significantly different, which implied that USP11 might serve a potential cancer-related biomarker (Fig. 3A). Preliminary analysis indicated that USP11 expression is closely associated with several cancer survival and prognosis, including low-grade glioma (LGG); liver hepatocellular carcinoma (LIHC), pancreatic cancer (PAAD), skin cutaneous melanoma (SKCM), and uveal melanoma (UVM) (Fig. 3B). The dysregulation of USP11 is associated with multiple diseases and pathological processes, and cancer therapies targeting USP11 have achieved expected clinical value and are gradually becoming an emerging therapeutic target for a variety of cancers, receiving attention from numerous researchers and scholars. In this review, we summarize the role of USP11 in different cancers and discuss the advantages and possibilities of targeting USP11 as a new therapeutic strategy for a variety of cancers (Table 3).

**Table 3:** The role of USP11 in cancer management.

Cancer types	Substrates	Effects	Role of USP11	Reference
Colorectal cancer	PPP1CA	USP11 facilitates growth and metastasis of colorectal cancer	Promoters	[78]
	IGF2BP3	USP11 promotes colorectal cancer proliferation and metastasis	Promoters	[79]
	VCP	USP11 induce resistance to 5-Fluorouracil in colorectal cancer via activating autophagy	Promoters	[34]
	P21	USP11 inhibits growth and metastasis of colorectal cancer	Inhibitor	[32]
	Cyclin D1	Circ-DDCK1 regulates USP11 via miR-132-3P to promote colorectal cancer progression	Promoters	[38]
Lung squamous cell carcinoma	ARID1A	Trim32/USP11 regulates ARID1A stability and the occurrence/inhibition of lung squamous cell carcinoma	Inhibitor	[85]
Breast cancer	PTEN	USP11 regulates PTEN to stabilize tumor suppression	Inhibitor	[92]
	XIAP	USP11 regulates XIAP stability to facilitate tumorigenesis of breast cancer	Promoters	[93]
	TGFBR2	USP11 triggers epithelial-to-mesenchymal transition and promotes invasion and proliferation of breast cancer	Promoters	[94]
	ERα/Cyclin D1	USP11 is identified as a novel transcriptional regulator of ERα in breast cancer	Promoters	[36]
	Non	USP11 as a predictive and prognostic factor following neoadjuvant therapy in breast cancer	Biomarker	[95]
Esophageal squamous cell carcinoma	ARID1A	Trim32/USP11 regulates ARID1A stability and the occurrence/inhibition of esophageal squamous cell carcinoma	Inhibitor	[85]
Ovarian cancer	BIP	USP11 promotes ovarian cancer chemoresistance of carboplatin	Promoters	[28]
	Snail	USP11 promotes epithelial-to-mesenchymal transition of ovarian cancer	Promoters	[99]
Prostate cancer	PTEN	USP11 regulates PTEN to stabilize tumor suppression	Inhibitor	[92]
Renal clear cell adenocarcinoma	VGLL4	USP11 functions as a tumor suppressor through stabilizing VGLL4	Inhibitor	[100]
Hepatocellular carcinoma	NF90	USP11 regulates NF90 to promote proliferation and metastasis in hepatocellular carcinoma	Promoters	[88]
	KLF4	USP11 facilitates the progression and sorafenib chemoresistance of hepatocellular carcinoma	Promoters	[30]
	E2F1	E2F1/USP11 positive feedback loop promotes hepatocellular carcinoma metastasis and inhibits autophagy by activating ERK/mTOR pathway	Promoters	[87]
	Non	USP11 serves as a marker of poor prognosis and promotes metastasis in hepatocellular carcinoma	Biomarker	[86]
Gastric cancer	RhoA	USP11 facilitates chemotherapy resistance of gastric cancer via RhoA and Ras-mediated signaling pathway	Promoters	[35]
Pancreatic cancer	BRCA2	Mitoxantrone targets USP11 and is a potent inhibitor of pancreatic cancer cell survival	Promoters	[89]
Melanoma	NONO	USP11 stabilizes NONO and promotes the proliferation of melanoma	Promoters	[107]
Skin squamous cell carcinoma	VCP/P97	USP11 is declined in skin squamous cell carcinoma and serve a potential tumor suppressor	Biomarker	[21]
Glioma	PML	USP11 stabilizes PML stability to control Notch-induced malignancy in brain tumors	Inhibitor	[33]
Non-small cell lung cancer	P21	USP11 inhibits the proliferation of non-small cell lung cancer dependent on p21 activity	Inhibitor	[32]
Osteosarcoma	RAE	USP11 promotes the proliferation in osteosarcoma U2OS cell	Promoters	[105]
	Non	USP11 as a survival-related differentially expressed gene	Biomarker	[111]
Cervical cancer	HPV-16E7	USP11 stabilizes HPV-16E7 to promote progression of cervical cancer	Promoters	[104]
Lymphoma	eIF4B	Activated USP11 promotes lymphomagenesis via stabilizing and deubiquitinating eIF4B	Promoters	[37]

### Gastrointestinal cancer

#### Colorectal cancer

Colorectal cancer is one of the most common malignancies among cancers, with ~104,270 newly diagnosed patients and 52980 deaths in 2021 according to the American Cancer Statistics [76]. There is no bias between male and female patients in terms of incidence and mortality. Multiple researches have shed light on the pathogenesis and factors associated with colorectal cancer, some of which have been applied to clinical treatment [77]. Recently, the role of USP11 in colorectal cancer has gradually emerged, Hongze Sun et al. [78] found that USP11 plays a central role in promoting the progression of colorectal cancer via stabilizing PPP1CA involved in ERK/MAPK signaling pathway. Weitong Zhang et al. [38] found USP11 promotes the proliferation and migration of colorectal cancer, and the Circ-DOCK1/MiR-132-3p axis further repressed USP11 transcription. Yayu Huang et al. [79] identified that USP11 was upregulated in colorectal cancer and facilitated the proliferation and metastasis by regulating the stability of IGF2BP3, which was also found to be related to liver cancer [80, 81], pancreatic cancer [82] and ovarian cancer [83, 84], implying that USP11-IGF2B3 might regulate the progression of more cancers. Tanggang Deng et al. [32] found that USP11 inhibits the proliferation of colorectal cancer via deubiquitinating and stabilizing P21, which depends on the intact function of P21. Hongze Sun et al. [34] further found that USP11 induced drug resistance to 5-Fluorouracil in colorectal cancer through activating autophagy by stabilizing VCP. The above results suggest that USP11 not only promotes or inhibits colorectal cancer, but also mediates chemotherapy resistance in colorectal cancer, and targeting USP11 may be an effective therapeutic strategy for colorectal cancer patients (Fig. 3). Further mechanisms and clinical applications require more research and discussion.

#### Squamous cell carcinoma

**Fig. 4:**
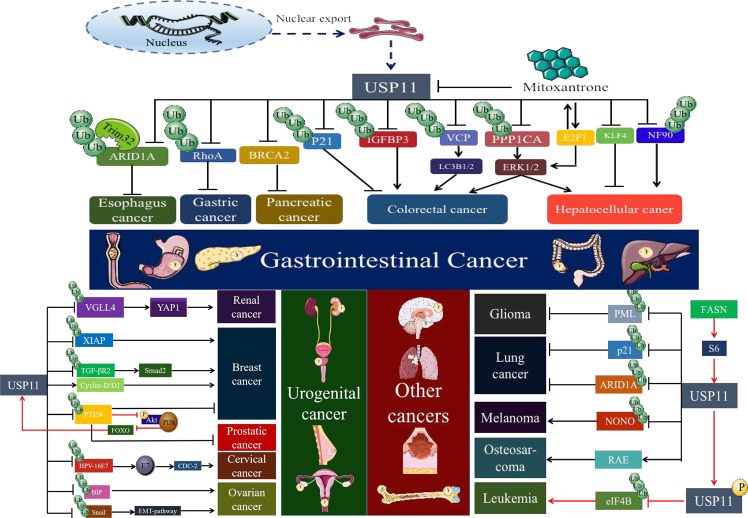
The role of USP11 in multiple cancers. 1. The role of USP11 in gastrointestinal tumors. USP11 facilitates the growth and metastasis of colorectal cancer via deubiquitinating and stabilizing PPPICA, IGF2BP3, and Cyclin D1, and promotes the chemoresistance to 5-Fluorouracil in colorectal cancer via deubiquitinating VCP-activated autophagy. In addition to its promotional effects, USP11 also inhibits the growth and metastasis of colorectal cancer. USP11 promotes the proliferation and metastasis of hepatocellular carcinoma by deubiquitinating and mediating NF90, KLF4 (mediating chemoresistance), and E2F1 (regulating the transcription of USP11). USP11 promotes chemoresistance in gastric cancer through RhoA and Ras-mediated signaling pathways. Mitoxantrone targets USP11 and is a potent inhibitor of pancreatic cancer cell survival. Trim32/USP11 regulates ARID1A stability and the occurrence or inhibition of esophageal squamous cell carcinoma. 2. The role of USP11 in genitourinary tumors. USP11 facilitates tumorigenesis, proliferation, and invasion of breast cancer via deubiquitinating XIAP, TGFBR2, and cyclin D1. USP11 also regulates PTEN to stabilize tumor suppression. USP11 facilitates epithelial-to-mesenchymal transition in ovarian cancer via deubiquitinating Snail, and promotes ovarian cancer chemoresistance of carboplatin by stabilizing BIP. USP11 significantly restrains the tumor progression via deubiquitinating and stabilizing PTEN (prostate cancer) and VGLL4 (Renal clear cell adenocarcinoma). USP11 also stabilizes HPV-16E7 to promote the progression of cervical cancer. 3. The role of USP11 in other cancers. USP11 facilitates the occurrence and progression of tumors via deubiquitinating and stabilizing NONO (melanoma), RAE (osteosarcoma), HPV-16E7 (cervical cancer), and EIF4B (promoting lymphoma by being phosphorylated). USP11 inhibits the occurrence and development of tumors by deubiquitinating ARID1A (lung squamous cell carcinoma), PML (glioma), and P21 (non-small cell lung cancer).

Squamous cell carcinoma is one of the most common aggressive epithelial malignancies, the occurrence and mechanism of which has attracted a lot of attention from researchers. Esophageal squamous cell carcinoma and lung squamous cell carcinoma are the most common types. Qingyu Luo et al. [85] found that the mutation rate and expression of the tumor suppressor ARID1A in squamous cell carcinoma was relatively low, which was inconsistent with other cancers. ARID1A significantly inhibits the proliferation and progression of squamous cell carcinoma via mediating with SDC2, WNT, Akt and Ras signaling pathways. The ubiquitinase Trim32 can ubiquitinate ARID1A and further promote its degradation, while USP11 inhibits the ubiquitination of ARID1A and maintains its cancer-inhibiting effect. The balance between Trim32 and USP11 maintains the activity and level of ARID1A to regulate the occurrence and progression of esophageal squamous cell carcinoma and lung squamous cell carcinoma (Fig. 4). Targeting Trim32/USP11/ARID1A/SDC2 might be a potential therapeutic strategy for patients with squamous cell carcinoma. As for the role of USP11 in the remaining squamous cell carcinoma, more researches are needed.

#### Hepatocellular carcinoma

The role of USP11 in hepatocellular carcinoma has attracted the attention of numerous scholar, USP11 might be an excellent biomarker and therapeutic target for lots of hepatocellular carcinoma patients. Sheng Zhang et al. [86] identified that USP11 served as a marker of poor prognosis and promoted metastasis in hepatocellular carcinoma. Lijun Qiao et al. [87] found that USP11 stabilized E2F1 which regulated USP11 transcription conversely, and the E2F1/USP11 formed a positive feedback loop to promote the proliferation and migration of HCC cells. Moreover, E2F1/USP11 further facilitates the progression of hepatocellular carcinoma by regulating the ERK/mTOR pathway to inhibit autophagy. Changmao Zhang et al. [88] identified that USP11 promoted the proliferation and metastasis of hepatocellular carcinoma via deubiquitinating NF90, which was also proved to promote the proliferation and angiogenesis of cervical cancer. The relationship between cervical cancer and USP11/NF90 remains to be further investigated. Furthermore, Heeyoung Yang et al. [30] identified that USP11 promotes the proliferation, tumorigenesis, and sorafenib-resistance of hepatocellular carcinoma via negatively regulating KLF4, which was known as a tumor suppressor factor in previous researches. They further found that USP11 was negatively related to different liver diseases, such as fatty liver and non-alcoholic fatty liver disease. These evidences further identify the critical role of USP11/KLF4 axis in the occurrence and development of different liver diseases, which might be a potential target for the treatment of various liver-origin diseases. Based on the above researches, we found that USP11 plays a promoting role in the progression of hepatocellular carcinoma and mediates chemoresistance, and targeting USP11 would significantly inhibit the progression and increase chemosensitivity in hepatocellular carcinoma (Fig. 4).

#### Gastric and pancreatic cancer

Previous researches have proved that USP11 is associated with other gastrointestinal cancers, such as gastric and pancreatic cancer. Hongfang Liu et al. [35] identified that the inhibition of USP11 sensitized gastric cancer to chemotherapy via suppressing RhoA and Ras-mediated signaling pathways, which implies that the inhibition of USP11 sensitized gastric cancer to chemotherapy (Fig. 4). Targeting USP11 is an excellent strategy for gastric cancer treatment and chemoresistance. Richard A. Burkhart et al. [89] found that mitoxantrone target USP11, a potential inhibitor of pancreatic cancer cell survival, implying that USP11 promotes the progression of pancreatic cancer and is a natural therapeutic target (Fig. 4). The research also provided a new therapeutic drug targeting USP11. All above results demonstrate that USP11 is related to the occurrence and progression of gastrointestinal cancer.

### Genitourinary cancer

#### Breast cancer

Breast cancer is the most common malignancy among female cancer patients. Based on cancer statistics in America 2021, there are about 281,550 new diagnosed breast cancer in female accounting for 30%, while ~43,600 deaths from breast cancer rating second cancer-resourcing death [76, 90]. There have been numerous researches on the mechanism of breast cancer occurrence and progression [91]. Recently, the relationship between USP11 and breast cancer has been identified and discussed, Mi Kyung Park et al. [92] found that USP11 is downregulated and functions as a mechanism of PTEN inactivation in the absence of PTEN genome, which restrains Akt signaling pathway activation and ultimately inhibits tumor proliferation. Zhuan Zhou et al. [93] identified that USP11 facilitates epithelial transformation of mammary epithelial cells and breast carcinogenesis via deubiquitination of XIAP. Intriguingly, USP11 was differentially expressed in the above studies, and different breast cancer subtypes and tumor microenvironments may be plausible explanations. Daniel A. Garcia et al. [94] found that USP11 enhanced TGFβ-induced epithelial–mesenchymal plasticity and breast cancer metastasis. Lisa Dwane et al. [36] identified USP11 as a novel transcriptional regulator of ERα in breast cancer through a functional genomic screen, and they found that USP11 could significantly inhibit the activity of ERα and the transcription of downstream target genes in response to estradiol stimulation. USP11 might be a potential therapeutic target for breast cancer patients with ERα mutations and chemotherapy resistance. Furthermore, Soley Bayraktar et al. [95] identified USP11 as a predictive and prognostic marker following neoadjuvant therapy for breast cancer. All above researches imply that USP11 not only significantly affects breast cancer progression but also mediates chemotherapy drugs resistance (Fig. 4). Although the complicated role of USP11 in the occurrence, development and drug resistance of breast cancer needs more and more rigorous researches. Targeting USP11 is an excellent strategy for different breast cancer subtypes, and USP11 also is a potential predictive and prognostic biomarker for breast cancer progression and drug resistance.

#### Ovarian cancer

Ovarian cancer is one of the most common malignancies in gynecology, high morbidity and mortality remain the most problematic issue for lots of patients with ovarian cancer. According to statistics in America, there are ~21,410 newly diagnosed ovarian cancer patients and 13,770 deaths from ovarian cancer in 2021 [76]. Previous researches have proved that DUBs are involved in the occurrence and progression of ovarian cancer [96–98]. Upregulated USP11 expression was found in ovarian cancer and related to poorer prognosis [99], USP11 promoted epithelial-to-mesenchymal transition by deubiquitinating snails, which finally facilitated the invasion and metastasis of ovarian cancer. Moreover, Xiaolin Zhu et al. [28] identified that USP11 promotes carboplatin-chemoresistance in ovarian cancer by stabilizing BIP dependent on its catalytic activity, and USP11 non-specific inhibitor mitoxantrone effectively increases the sensitivity of ovarian cancer to carboplatin chemotherapy. Targeting USP11-BIP axis might be a therapeutic strategy to improve the chemosensitivity of patients with ovarian cancer. All above researches show that USP11 is a typical oncogenic factor that might be a potential therapeutic target for ovarian cancer patients (Fig. 4). Targeting USP11 might increase the survival and chemosensitivity of ovarian cancer, and USP11 specific/non-specific inhibitors will surely be applied to clinical treatment soon.

#### Prostate and renal clear cell cancer

The clinical significance of USP11 has also been demonstrated in urinary system cancers. Based on the current research, it has been established that USP11 plays an inhibitory role in the occurrence and progression of urological cancers. Mi Kyung Park et al. [92] also found that USP11 is downregulated in prostate cancer, related to PTEN instability and poorer prognosis, the USP11-PTEN-PI3K-AKT loop signaling pathway regulates the occurrence and progression of prostate cancer. In addition, USP11 not only regulates the status of prostate cancer via stabilizing PTEN, but also acts as a carrier of cell density to control the physiological dose of PTEN protein. Encheng Zhang et al. [100] found that USP11 functions as a tumor suppressor through deubiquitinating and stabilizing VGLL4 protein, a typical transcription repressor that inhibits the YAP signaling pathway to further restrain the proliferation of renal clear cell adenocarcinoma cells 786-O. Further experiments and tissue validation will be conducted in future researches, and we have enough reasons to predict that USP11 might be a potential inhibitor of urological cancers (Fig. 4).

#### Cervical cancer

Cervical cancer is one of the most common malignancies in gynecology, and its incidence has gradually declined with the promotion and application of HPV vaccine [101]. The 5-year survival rate of these patients with metastatic cervical cancer remains low, and the underlying mechanisms still need further research and investigation [102, 103]. USP11 promotes cervical cancer progression and proliferation through deubiquitination and stabilization of HPV-16E7, subsequently affecting the biological function of E7 as well as the HPV-16E7 contribution to cellular transformation [104] (Fig. 4). This article merely introduces USP11 into cervical cancer, and the more complicated role and function of USP11 in cervical cancer will be gradually revealed. The significant inhibition of cervical cancer proliferation and progression by declining USP11 implies that targeting USP11 is a new and viable therapeutic manner for many cervical cancer patients.

### Other cancers

#### Melanoma

Melanoma is a highly malignant tumor type with a high mortality. In the United States, there are about 106,110 newly diagnosed melanoma patients and ~7180 deaths from melanoma in 2021 [76]. Although much achievement has been accomplished with medical and pharmaceutical advances, the survival of patients with metastatic melanoma remains low [105, 106]. Peifu Feng et al. [107] found a novel mechanism by which USP11 facilitates the proliferation of melanoma via deubiquitinating NONO, which is upregulated in melanoma and related to poor prognosis. This implies that targeting USP11/NONO might be a therapeutic strategy for melanoma, providing a new direction for clinical treatment options. Besides melanoma, Palak Shah et al. [21] found that USP11 was related to different skin tumors and that USP11 levels would gradually decrease from normal skin tissue, actinic keratosis, to squamous cell carcinoma. The expression of USP11 also gradually decreased in UVB-irradiated hormonal mice. Decreased USP11 promotes premature separation of X4 from damage site to postpone DNA damage repair via VCP/P97. These results imply that USP11 might be a tumor suppressor of skin cancers, and its downregulation is one of the early events in skin tumor (Fig. 4). The contradictory role of USP11 in skin tumors might be a disturbing consequence of DNA damage. The specific mechanism and reasons need more efforts and researches.

#### Glioma

The significance of USP11 in glioma tumorigenesis has been demonstrated by its positive regulation on PML, which is downregulated in majority of cancers and played inhibiting cancer role [108, 109]. Hsin-Chieh Wu et al. [33] found that USP11 inhibits the occurrence and progression of glioma via deubiquitinating and stabilizing PML. Further research identified that the transcription of USP11 is restrained by Notch-induced Hey, which is core inhibitor in USP11 promoter region. USP11 not only restrains the proliferation and invasion of glioblastoma multiforme but also further deterioration of glioma-initiating cells. Increasing USP11 or targeting Notch signaling pathway might be a feasible therapeutic strategy for numerous glioma patients (Fig. 4).

#### Osteosarcoma

The critical value of USP11 in the occurrence and progression of osteosarcoma has been identified via its deubiquitylation and stabilization of RAE1. Anna Stockum et al. [110] found that USP11 interacts with RAE1 and facilitates the proliferation of osteosarcoma cell U2OS via deubiquitinating RAE1. They further found that USP11 is associated with the mitotic spindle and does not regulate SAC inactivation, but controls ubiquitination of RAE1 at the mitotic spindle, thereby functionally modulating its interaction with Nuclear Mitotic Apparatus protein (NuMA). Furthermore, Emel Rothzerg et al. [111] identified USP11 as a differentially expressed gene associated with survival by screening a database of target osteosarcomas. Although the results of the above researches are based on cellular aspects and databases, the relationship between USP11 and osteosarcoma is undoubtedly accurate. Targeting USP11 as a therapeutic strategy for osteosarcoma is feasible and more experiments are needed to further verification (Fig. 4).

#### Non-small cell lung cancer and lymphoma

These researches also provide a new therapeutic drug for targeting USP11. Tanggang Deng et al. [32] identified that USP11 inhibits the proliferation of non-small cell lung cancer via deubiquitinating and stabilizing P21 (Fig. 4). Bandish Kapadia et al. [37] found that phosphorylation of USP11 stabilizes and deubiquitinates transcription factor EIF4B and further promotes the progression and malignancy of diffuse large B-cell lymphoma, USP11 is phosphorylated by FASN-induced S6 kinase, which also suggests that the status of lymphoma is affected by altered lipid metabolism (Fig. 4). All above results demonstrate that USP11 is related to the occurrence and progression of multiple cancers, targeting USP11 or using ono-specific USP11 inhibitor mitoxantrone is a feasible and effective therapeutic approach for various cancer treatment.

## USP11 in other diseases

In addition to the critical role of USP11 in multiple cancers, it regulates the occurrence and status of different diseases. The balance between USP11 and other ubiquitinase maintain the expression and activation status of critical proteins of cancer-related signaling pathways. Xiuqing Zhang et al. [112] identified that USP11 significantly restrained the transcription of KLF2- NF-κB signaling pathway via stabilizing p53 to facilitate intracerebral hemorrhage-induced pro-inflammatory factors release and neurological impairment. Targeting USP11/P53/KLF2/ NF-κB might be a novel anti-inflammatory approach for the treatment of intracerebral hemorrhage. In addition, Zhiwei Xu et al. [113] also identified that USP11 was associated with neuronal apoptosis following intracerebral hemorrhage. Shang-Yin Chiang et al. [18] found that USP11 controlled cortical neurogenesis and neuronal migration through stabilizing Sox11. Roman Istomine et al. [114] found that USP11 potentiates TGF-β signaling in CD4^+^ T cells to facilitate Foxp3^+^ regulatory T and Th17 cell differentiation. Jing Zhao et al. [115] identified that the balance of USP11 and Nedd4L maintained the stability and status of LPA1, and that USP11 facilitated the pro-inflammatory effects and lung injury via deubiquitinating LPA1. This article provides a potential target for the development of anti-inflammatory molecules to lessen lung injury. The role of USP11 is also critical and obvious during infection, Tsai-Ling Liao et al. [116] identified that USP11 inhibits influenza A virus RNA replication via deubiquitinating NP, which can be utilized to manipulate antiviral therapeutic purpose. The effect of USP11 in other diseases is not merely mentioned above, but the role of deubiquitinase determines its complication. Targeting USP11 might be a potential and effective therapeutic measure for multiple diseases and disorders.

## Targeting USP11 for cancer clinical treatment

USP11 has an important impact on the development and progression of multiple cancers, and previous studies have shown that targeting USP11 can significantly inhibit the proliferation and migration of multiple cancers. In some articles, USP11 plays an inhibitory role in the progression of cancers such as glioma and renal clear cell adenocarcinoma, and increasing USP11 transcription or applying external USP11 may be a viable strategy for cancer therapy. Currently, there are no USP11-specific inhibitors targeting USP11 therapy, and targeting the USP11 catalytic center may be the direction and mechanism to design USP11-specific inhibitors. Previous researches identified that mitoxantrone, used for the treatment of acute myeloid leukemia [117, 118], multiple sclerosis [119, 120], and hormone-resistant prostate cancer [121, 122], is a non-specific USP11 inhibitor [89, 114, 115, 123]. Mitoxantrone significantly restrains the activity and subsequent effects of USP11, and higher USP11 level further facilitates the inhibitory efficiency of mitoxantrone. Targeting USP11 USP domain small molecule compounds might be the most mainstream direction for future clinical treatment.

USP11 expression can also be regulated via regulating the activity and levels of related transcription factors to increase or inhibit USP11 transcription, such as inhibiting Notch-induced Hey1 to increase USP11 transcription to restrain glioma progression. Structural studies have demonstrated that USP11 interacts with USP7, which has been identified to synergistically regulate the progression and status of multiple diseases [48, 49]. Several reported USP7 inhibitor, including HBX 41108 [124], HBX19818 [125], HBX28258 [125], and P022077 [126] might be effective for the inhibition of USP11, which needs further verification. Structural studies have been performed on only a handful of DUBs. Therefore, these challenges will provide new opportunities for drug discovery for clinically relevant DUBs, including USP11. Based on the current researches, it is undoubtable that targeting USP11 is a feasible and effective therapeutic strategy, especially for multiple malignancies and chemotherapy-resistant cancers.

## Conclusion and future perspective

Ubiquitination and deubiquitylation are among the most common post-translational modifications involved in a variety of physiological and pathological processes, such as regulating protein stability, cellular pathways, cancer-related signaling, DNA damage repair, and response to various stresses. USP11 has been shown to play a critical role in a variety of cancers and chemotherapy resistance, which is a potential target for cancer therapy. Currently, the non-specific USP11 inhibitor mitoxantrone has shown the considerable obvious USP11 inhibitory effect, and the specific USP11 inhibitors remain to be further investigated and developed. Targeting the catalytic structural domain of USP11 is the main target for the design of specific USP11 inhibitory molecules. Regardless, USP11 may be a potentially beneficial therapeutic target for cancer therapy and chemotherapy resistance. Therefore, its clinical relevance and application should be extensively explored, investigated, and evaluated.

## References

[1] Zhao BS, Roundtree IA, He C (2017). Post-transcriptional gene regulation by mRNA modifications. Nat Rev Mol Cell Biol.

[2] Spoel SH, Tada Y, Loake GJ (2010). Post-translational protein modification as a tool for transcription reprogramming. N. Phytologist.

[3] Rape M (2018). Ubiquitylation at the crossroads of development and disease. Nat Rev Mol Cell Biol.

[4] Popovic D, Vucic D, Dikic I (2014). Ubiquitination in disease pathogenesis and treatment. Nat Med.

[5] Doerr A (2018). Comprehensive mapping of ubiquitination. Nat Methods.

[6] Buetow L, Huang DT (2016). Structural insights into the catalysis and regulation of E3 ubiquitin ligases. Nat Rev Mol Cell Biol.

[7] Liu B, Ruan J, Chen M, Li Z, Manjengwa G, Schlüter D (2022). Deubiquitinating enzymes (DUBs): decipher underlying basis of neurodegenerative diseases. Mol Psychiatry.

[8] David R (2011). DUBs’ key to selectivity. Nat Rev Mol Cell Biol.

[9] He M, Zhou Z, Wu G, Chen Q, Wan Y (2017). Emerging role of DUBs in tumor metastasis and apoptosis: therapeutic implication. Pharmacol Therapeutics.

[10] Chen D, Ning Z, Chen H, Lu C, Liu X, Xia T (2020). An integrative pan-cancer analysis of biological and clinical impacts underlying ubiquitin-specific-processing proteases. Oncogene.

[11] Young MJ, Hsu KC, Lin TE, Chang WC, Hung JJ (2019). The role of ubiquitin-specific peptidases in cancer progression. J Biomed Sci.

[12] Akimov V, Barrio-Hernandez I, Hansen SVF, Hallenborg P, Pedersen AK, Bekker-Jensen DB (2018). UbiSite approach for comprehensive mapping of lysine and N-terminal ubiquitination sites. Nat Struct Mol Biol.

[13] Wiener R, Zhang X, Wang T, Wolberger C (2012). The mechanism of OTUB1-mediated inhibition of ubiquitination. Nature.

[14] Carroll EC, Greene ER, Martin A, Marqusee S (2020). Site-specific ubiquitination affects protein energetics and proteasomal degradation. Nat Chem Biol.

[15] Ideguchi H, Ueda A, Tanaka M, Yang J, Tsuji T, Ohno S (2002). Structural and functional characterization of the USP11 deubiquitinating enzyme, which interacts with the RanGTP-associated protein RanBPM. Biochemical J.

[16] Harper S, Gratton HE, Cornaciu I, Oberer M, Scott DJ, Emsley J (2014). Structure and catalytic regulatory function of ubiquitin specific protease 11 N-terminal and ubiquitin-like domains. Biochemistry.

[17] Spiliotopoulos A, Blokpoel Ferreras L, Densham RM, Caulton SG, Maddison BC, Morris JR (2019). Discovery of peptide ligands targeting a specific ubiquitin-like domain-binding site in the deubiquitinase USP11. J Biol Chem.

[18] Chiang SY, Wu HC, Lin SY, Chen HY, Wang CF, Yeh NH, et al. Usp11 controls cortical neurogenesis and neuronal migration through Sox11 stabilization. Sci Adv. 2021;7:eabc6093. 10.1126/sciadv.abc6093 PMC7880594 33579706

[19] Brandau O, Nyakatura G, Jedele KB, Platzer M, Achatz H, Ross M (1998). UHX1 and PCTK1: precise characterisation and localisation within a gene-rich region in Xp11.23 and evaluation as candidate genes for retinal diseases mapped to Xp21.1-p11.2. Eur J Hum Genet: EJHG.

[20] Ting X, Xia L, Yang J, He L, Si W, Shang Y (2019). USP11 acts as a histone deubiquitinase functioning in chromatin reorganization during DNA repair. Nucleic Acids Res.

[21] Shah P, Qiang L, Yang S, Soltani K, He YY (2017). Regulation of XPC deubiquitination by USP11 in repair of UV-induced DNA damage. Oncotarget.

[22] Wiltshire TD, Lovejoy CA, Wang T, Xia F, O’Connor MJ, Cortez D (2010). Sensitivity to poly(ADP-ribose) polymerase (PARP) inhibition identifies ubiquitin-specific peptidase 11 (USP11) as a regulator of DNA double-strand break repair. J Biol Chem.

[23] Schoenfeld AR, Apgar S, Dolios G, Wang R, Aaronson SA (2004). BRCA2 is ubiquitinated in vivo and interacts with USP11, a deubiquitinating enzyme that exhibits prosurvival function in the cellular response to DNA damage. Mol Cell Biol.

[24] Perry M, Biegert M, Kollala SS, Mallard H, Su G, Kodavati M (2021). USP11 mediates repair of DNA-protein cross-links by deubiquitinating SPRTN metalloprotease. J Biol Chem.

[25] Sun W, Tan X, Shi Y, Xu G, Mao R, Gu X (2010). USP11 negatively regulates TNFalpha-induced NF-kappaB activation by targeting on IkappaBalpha. Cell Signal.

[26] Yamaguchi T, Kimura J, Miki Y, Yoshida K (2007). The deubiquitinating enzyme USP11 controls an IkappaB kinase alpha (IKKalpha)-p53 signaling pathway in response to tumor necrosis factor alpha (TNFalpha). J Biol Chem.

[27] Li N, Feng L, Han HQ, Yuan J, Qi XK, Lian YF (2016). A novel Smac mimetic APG-1387 demonstrates potent antitumor activity in nasopharyngeal carcinoma cells by inducing apoptosis. Cancer Lett.

[28] Zhu X, Zhang Y, Luo Q, Wu X, Huang F, Shu T (2021). The deubiquitinase USP11 promotes ovarian cancer chemoresistance by stabilizing BIP. Signal Transduct Target Ther.

[29] Al-Salihi MA, Herhaus L, Macartney T, Sapkota GP (2012). USP11 augments TGFβ signalling by deubiquitylating ALK5. Open Biol.

[30] Yang H, Park D, Ryu J, Park T (2021). USP11 degrades KLF4 via its deubiquitinase activity in liver diseases. J Cell Mol Med.

[31] Wang D, Zhao J, Li S, Wei J, Nan L, Mallampalli RK (2018). Phosphorylated E2F1 is stabilized by nuclear USP11 to drive Peg10 gene expression and activate lung epithelial cells. J Mol Cell Biol.

[32] Deng T, Yan G, Song X, Xie L, Zhou Y, Li J (2018). Deubiquitylation and stabilization of p21 by USP11 is critical for cell-cycle progression and DNA damage responses. Proc Natl Acad Sci USA.

[33] Wu HC, Lin YC, Liu CH, Chung HC, Wang YT, Lin YW (2014). USP11 regulates PML stability to control Notch-induced malignancy in brain tumours. Nat Commun.

[34] Sun H, Wang R, Liu Y, Mei H, Liu X, Peng Z (2021). USP11 induce resistance to 5-fluorouracil in colorectal cancer through activating autophagy by stabilizing VCP. J Cancer.

[35] Liu H, Liu M, He B, Li Q. Inhibition of USP11 sensitizes gastric cancer to chemotherapy via suppressing RhoA and Ras-mediated signaling pathways. Clin Res Hepatol Gastroenterol. 2021;46**:**101779. 10.1016/j.clinre.2021.101779 34332125

[36] Dwane L, O’Connor AE, Das S, Moran B, Mulrane L, Pinto-Fernandez A (2020). A functional genomic screen identifies the deubiquitinase USP11 as a novel transcriptional regulator of ERα in breast cancer. Cancer Res.

[37] Kapadia B, Nanaji NM, Bhalla K, Bhandary B, Lapidus R, Beheshti A (2018). Fatty Acid Synthase induced S6Kinase facilitates USP11-eIF4B complex formation for sustained oncogenic translation in DLBCL. Nat Commun.

[38] Zhang W, Wang Z, Cai G, Huang P (2021). Circ_DOCK1 regulates USP11 through miR-132-3p to control colorectal cancer progression. World J surgical Oncol.

[39] Mansour MA (2018). Ubiquitination: friend and foe in cancer. Int J Biochem Cell Biol.

[40] Sun T, Liu Z, Yang Q (2020). The role of ubiquitination and deubiquitination in cancer metabolism. Mol Cancer.

[41] Qi SM, Cheng G, Cheng XD, Xu Z, Xu B, Zhang WD (2020). Targeting USP7-mediated deubiquitination of MDM2/MDMX-p53 pathway for cancer therapy: are we there yet?. Front Cell Develop Biol.

[42] Zhao H, Wang Y, Yang C, Zhou J, Wang L, Yi K (2020). EGFR-vIII downregulated H2AZK4/7AC though the PI3K/AKT-HDAC2 axis to regulate cell cycle progression. Clin Transl Med.

[43] Choudhary C, Kumar C, Gnad F, Nielsen ML, Rehman M, Walther TC (2009). Lysine acetylation targets protein complexes and co-regulates major cellular functions. Science.

[44] Olsen JV, Vermeulen M, Santamaria A, Kumar C, Miller ML, Jensen LJ (2010). Quantitative phosphoproteomics reveals widespread full phosphorylation site occupancy during mitosis. Sci Signal.

[45] Rigbolt KT, Prokhorova TA, Akimov V, Henningsen J, Johansen PT, Kratchmarova I (2011). System-wide temporal characterization of the proteome and phosphoproteome of human embryonic stem cell differentiation. Sci Signal.

[46] Dephoure N, Zhou C, Villén J, Beausoleil SA, Bakalarski CE, Elledge SJ (2008). A quantitative atlas of mitotic phosphorylation. Proc Natl Acad Sci USA.

[47] Bian Y, Song C, Cheng K, Dong M, Wang F, Huang J (2014). An enzyme assisted RP-RPLC approach for in-depth analysis of human liver phosphoproteome. J Proteom.

[48] Georges A, Marcon E, Greenblatt J, Frappier L (2018). Identification and characterization of USP7 targets in cancer cells. Sci Rep.

[49] Maertens GN, El Messaoudi-Aubert S, Elderkin S, Hiom K, Peters G (2010). Ubiquitin-specific proteases 7 and 11 modulate Polycomb regulation of the INK4a tumour suppressor. EMBO J.

[50] Yuan L, Yuan P, Yuan H, Wang Z, Run Z, Chen G (2017). miR-542-3p inhibits colorectal cancer cell proliferation, migration and invasion by targeting OTUB1. Am J Cancer Res.

[51] Zhou Y, Wu J, Fu X, Du W, Zhou L, Meng X (2014). OTUB1 promotes metastasis and serves as a marker of poor prognosis in colorectal cancer. Mol Cancer.

[52] Liao Y, Wu N, Wang K, Wang M, Wang Y, Gao J (2020). OTUB1 promotes progression and proliferation of prostate cancer via deubiquitinating and stabling cyclin E1. Front Cell Develop Biol.

[53] Iglesias-Gato D, Chuan YC, Jiang N, Svensson C, Bao J, Paul I (2015). OTUB1 de-ubiquitinating enzyme promotes prostate cancer cell invasion in vitro and tumorigenesis in vivo. Mol Cancer.

[54] Karunarathna U, Kongsema M, Zona S, Gong C, Cabrera E, Gomes AR (2016). OTUB1 inhibits the ubiquitination and degradation of FOXM1 in breast cancer and epirubicin resistance. Oncogene.

[55] Baietti MF, Simicek M, Abbasi Asbagh L, Radaelli E, Lievens S, Crowther J (2016). OTUB1 triggers lung cancer development by inhibiting RAS monoubiquitination. EMBO Mol Med.

[56] Xie JJ, Guo QY, Jin JY, Jin D (2019). SP1-mediated overexpression of lncRNA LINC01234 as a ceRNA facilitates non-small-cell lung cancer progression via regulating OTUB1. J Cell Physiol.

[57] Ma A, Tang M, Zhang L, Wang B, Yang Z, Liu Y (2019). USP1 inhibition destabilizes KPNA2 and suppresses breast cancer metastasis. Oncogene.

[58] Cui SZ, Lei ZY, Guan TP, Fan LL, Li YQ, Geng XY (2020). Targeting USP1-dependent KDM4A protein stability as a potential prostate cancer therapy. Cancer Sci.

[59] Dai X, Lu L, Deng S, Meng J, Wan C, Huang J (2020). USP7 targeting modulates anti-tumor immune response by reprogramming tumor-associated macrophages in lung cancer. Theranostics.

[60] Grunblatt E, Wu N, Zhang H, Liu X, Norton JP, Ohol Y (2020). MYCN drives chemoresistance in small cell lung cancer while USP7 inhibition can restore chemosensitivity. Genes Dev.

[61] Rong Z, Zhu Z, Cai S, Zhang B (2020). Knockdown of USP8 inhibits the growth of lung cancer cells. Cancer Manag Res.

[62] Sun J, Shen D, Zheng Y, Ren H, Liu H, Chen X (2020). USP8 inhibitor suppresses HER-2 positive gastric cancer cell proliferation and metastasis via the PI3K/AKT signaling pathway. OncoTargets Ther.

[63] Peng Y, Liao Q, Tan W, Peng C, Hu Z, Chen Y (2019). The deubiquitylating enzyme USP15 regulates homologous recombination repair and cancer cell response to PARP inhibitors. Nat Commun.

[64] Chen LL, Smith MD, Lv L, Nakagawa T, Li Z, Sun SC, et al. USP15 suppresses tumor immunity via deubiquitylation and inactivation of TET2. Sci Adv. 2020;6:eabc9730. 10.1126/sciadv.abc9730 PMC7500937 32948596

[65] Wang Y, Sun Q, Mu N, Sun X, Wang Y, Fan S (2020). The deubiquitinase USP22 regulates PD-L1 degradation in human cancer cells. Cell Commun Signal.

[66] Li J, Yuan S, Norgard RJ, Yan F, Yamazoe T, Blanco A (2020). Tumor cell-intrinsic USP22 suppresses antitumor immunity in pancreatic cancer. Cancer Immunol Res.

[67] McCann JJ, Vasilevskaya IA, Poudel Neupane N, Shafi AA, McNair C, Dylgjeri E (2020). USP22 functions as an oncogenic driver in prostate cancer by regulating cell proliferation and DNA repair. Cancer Res.

[68] Zhang B, Yang C, Wang R, Wu J, Zhang Y, Liu D (2020). OTUD7B suppresses Smac mimetic-induced lung cancer cell invasion and migration via deubiquitinating TRAF3. J Exp Clin Cancer Res.

[69] Lei S, He Z, Chen T, Guo X, Zeng Z, Shen Y (2019). Long noncoding RNA 00976 promotes pancreatic cancer progression through OTUD7B by sponging miR-137 involving EGFR/MAPK pathway. J Exp Clin Cancer Res.

[70] Tang J, Wu Z, Tian Z, Chen W, Wu G (2021). OTUD7B stabilizes estrogen receptor α and promotes breast cancer cell proliferation. Cell Death Dis.

[71] Liu X, Zhang X, Peng Z, Li C, Wang Z, Wang C (2020). Deubiquitylase OTUD6B governs pVHL stability in an enzyme-independent manner and suppresses hepatocellular carcinoma metastasis. Adv Sci.

[72] Yoon CI, Ahn SG, Bae SJ, Shin YJ, Cha C, Park SE (2019). High A20 expression negatively impacts survival in patients with breast cancer. PLoS ONE.

[73] Shi Y, Wang X, Wang J, Wang X, Zhou H, Zhang L (2021). The dual roles of A20 in cancer. Cancer Lett.

[74] Zhang Z, Du J, Wang S, Shao L, Jin K, Li F (2019). OTUB2 promotes cancer metastasis via hippo-independent activation of YAP and TAZ. Mol Cell.

[75] Li J, Cheng D, Zhu M, Yu H, Pan Z, Liu L (2019). OTUB2 stabilizes U2AF2 to promote the Warburg effect and tumorigenesis via the AKT/mTOR signaling pathway in non-small cell lung cancer. Theranostics.

[76] Siegel RL, Miller KD, Fuchs HE, Jemal A (2021). Cancer Statistics, 2021. CA: Cancer J Clin.

[77] Zielińska A, Włodarczyk M, Makaro A, Sałaga M, Fichna J (2021). Management of pain in colorectal cancer patients. Crit Rev Oncol/Hematol.

[78] Sun H, Ou B, Zhao S, Liu X, Song L, Liu X (2019). USP11 promotes growth and metastasis of colorectal cancer via PPP1CA-mediated activation of ERK/MAPK signaling pathway. EBioMedicine.

[79] Huang YY, Zhang CM, Dai YB, Lin JG, Lin N, Huang ZX (2021). USP11 facilitates colorectal cancer proliferation and metastasis by regulating IGF2BP3 stability. Am J Transl Res.

[80] Li Z, Zhang J, Liu X, Li S, Wang Q, Di C (2018). The LINC01138 drives malignancies via activating arginine methyltransferase 5 in hepatocellular carcinoma. Nat Commun.

[81] Yang J, Qin T, Liu S, Tang H, Liu M, Wang Q (2020). Interaction analysis of miR-1275/IGF2BP1/IGF2BP3 with the susceptibility to hepatocellular carcinoma. Biomark Med.

[82] Cui XH, Hu SY, Zhu CF, Qin XH (2020). Expression and prognostic analyses of the insulin-like growth factor 2 mRNA binding protein family in human pancreatic cancer. BMC Cancer.

[83] Liu H, Zeng Z, Afsharpad M, Lin C, Wang S, Yang H (2019). Overexpression of IGF2BP3 as a potential oncogene in ovarian clear cell carcinoma. Front Oncol.

[84] Köbel M, Xu H, Bourne PA, Spaulding BO, Shih Ie M, Mao TL (2009). IGF2BP3 (IMP3) expression is a marker of unfavorable prognosis in ovarian carcinoma of clear cell subtype. Mod Pathol.

[85] Luo Q, Wu X, Nan Y, Chang W, Zhao P, Zhang Y (2020). TRIM32/USP11 balances ARID1A stability and the oncogenic/tumor-suppressive status of squamous cell carcinoma. Cell Rep.

[86] Zhang S, Xie C, Li H, Zhang K, Li J, Wang X (2018). Ubiquitin-specific protease 11 serves as a marker of poor prognosis and promotes metastasis in hepatocellular carcinoma. Lab Investig.

[87] Qiao L, Zhang Q, Sun Z, Liu Q, Wu Z, Hu W (2021). The E2F1/USP11 positive feedback loop promotes hepatocellular carcinoma metastasis and inhibits autophagy by activating ERK/mTOR pathway. Cancer Lett.

[88] Zhang C, Xie C, Wang X, Huang Y, Gao S, Lu J (2020). Aberrant USP11 expression regulates NF90 to promote proliferation and metastasis in hepatocellular carcinoma. Am J Cancer Res.

[89] Burkhart RA, Peng Y, Norris ZA, Tholey RM, Talbott VA, Liang Q (2013). Mitoxantrone targets human ubiquitin-specific peptidase 11 (USP11) and is a potent inhibitor of pancreatic cancer cell survival. Mol Cancer Res.

[90] DeSantis C, Siegel R, Bandi P, Jemal A (2011). Breast cancer statistics, 2011. CA: Cancer J Clin.

[91] Tray N, Taff J, Adams S (2019). Therapeutic landscape of metaplastic breast cancer. Cancer Treat Rev.

[92] Park MK, Yao Y, Xia W, Setijono SR, Kim JH, Vila IK (2019). PTEN self-regulates through USP11 via the PI3K-FOXO pathway to stabilize tumor suppression. Nat Commun.

[93] Zhou Z, Luo A, Shrivastava I, He M, Huang Y, Bahar I (2017). Regulation of XIAP turnover reveals a role for USP11 in promotion of tumorigenesis. EBioMedicine.

[94] Garcia DA, Baek C, Estrada MV, Tysl T, Bennett EJ, Yang J (2018). USP11 enhances TGFβ-induced epithelial-mesenchymal plasticity and human breast cancer metastasis. Mol Cancer Res.

[95] Bayraktar S, Gutierrez Barrera AM, Liu D, Pusztai L, Litton J, Valero V (2013). USP-11 as a predictive and prognostic factor following neoadjuvant therapy in women with breast cancer. Cancer J.

[96] Coughlin K, Anchoori R, Iizuka Y, Meints J, MacNeill L, Vogel RI (2014). Small-molecule RA-9 inhibits proteasome-associated DUBs and ovarian cancer in vitro and in vivo via exacerbating unfolded protein responses. Clin Cancer Res.

[97] Lei X, Li X, Chen H, Liu Z (2020). USP48 sustains chemoresistance and metastasis in ovarian cancer. Curr Cancer Drug Targets.

[98] Zhou H, Liu Y, Zhu R, Ding F, Cao X, Lin D (2018). OTUB1 promotes esophageal squamous cell carcinoma metastasis through modulating Snail stability. Oncogene.

[99] Wang W, Wang J, Yan H, Zhang K, Liu Y (2019). Upregulation of USP11 promotes epithelial-to-mesenchymal transition by deubiquitinating Snail in ovarian cancer. Oncol Rep.

[100] Zhang E, Shen B, Mu X, Qin Y, Zhang F, Liu Y (2016). Ubiquitin-specific protease 11 (USP11) functions as a tumor suppressor through deubiquitinating and stabilizing VGLL4 protein. Am J Cancer Res.

[101] Wang R, Pan W, Jin L, Huang W, Li Y, Wu D (2020). Human papillomavirus vaccine against cervical cancer: opportunity and challenge. Cancer Lett.

[102] Roden RBS, Stern PL (2018). Opportunities and challenges for human papillomavirus vaccination in cancer. Nat Rev Cancer.

[103] Markowitz LE, Naleway AL, Lewis RM, Crane B, Querec TD, Weinmann S (2019). Declines in HPV vaccine type prevalence in women screened for cervical cancer in the United States: evidence of direct and herd effects of vaccination. Vaccine.

[104] Lin CH, Chang HS, Yu WC (2008). USP11 stabilizes HPV-16E7 and further modulates the E7 biological activity. J Biol Chem.

[105] Siddiqui MF, Kim MM (2021). SIRT7 gene knockout using CRISPR/Cas9 system enhances melanin production in the melanoma cells. Biochimica et Biophysica Acta Mol Basis Dis.

[106] Song M, Xia W, Tao Z, Zhu B, Zhang W, Liu C (2021). Self-assembled polymeric nanocarrier-mediated co-delivery of metformin and doxorubicin for melanoma therapy. Drug Deliv.

[107] Feng P, Li L, Dai J, Zhou L, Liu J, Zhao J (2021). The regulation of NONO by USP11 via deubiquitination is linked to the proliferation of melanoma cells. J Cell Mol Med.

[108] Zhang ML, Fang JP (2021). Partial multi-label learning via credible label elicitation. IEEE Trans Pattern Anal Mach Intell.

[109] Astolfi A, Masetti R, Indio V, Bertuccio SN, Messelodi D, Rampelli S (2021). Torque Teno Mini Virus as a cause of childhood acute promyelocytic leukemia lacking PML/RARA fusion. Blood.

[110] Stockum A, Snijders AP, Maertens GN (2018). USP11 deubiquitinates RAE1 and plays a key role in bipolar spindle formation. PLoS ONE.

[111] Rothzerg E, Xu J, Wood D, Kõks S. 12 Survival-related differentially expressed genes based on the TARGET-osteosarcoma database. Exp Biol Med. 2021;246:2072–81. 10.1177/15353702211007410 PMC8524768 33926256

[112] Zhang X, Liu T, Xu S, Gao P, Dong W, Liu W (2021). A pro-inflammatory mediator USP11 enhances the stability of p53 and inhibits KLF2 in intracerebral hemorrhage. Mol Ther Methods Clin Dev.

[113] Xu Z, Li X, Chen J, Zhao J, Wang J, Ji Y (2016). USP11, deubiquitinating enzyme, associated with neuronal apoptosis following intracerebral hemorrhage. J Mol Neurosci.

[114] Istomine R, Alvarez F, Almadani Y, Philip A, Piccirillo CA (2019). The deubiquitinating enzyme ubiquitin-specific peptidase 11 potentiates TGF-β signaling in CD4(+) T cells to facilitate Foxp3(+) regulatory T and T(H)17 cell differentiation. J Immunol.

[115] Zhao J, Wei J, Dong S, Bowser RK, Zhang L, Jacko AM (2016). Destabilization of lysophosphatidic acid receptor 1 reduces cytokine release and protects against lung injury. EBioMedicine.

[116] Liao TL, Wu CY, Su WC, Jeng KS, Lai MM (2010). Ubiquitination and deubiquitination of NP protein regulates influenza A virus RNA replication. EMBO J.

[117] Amadori S, Arcese W, Isacchi G, Meloni G, Petti MC, Monarca B (1991). Mitoxantrone, etoposide, and intermediate-dose cytarabine: an effective and tolerable regimen for the treatment of refractory acute myeloid leukemia. J Clin Oncol.

[118] Christian S, Arain S, Patel P, Khan I, Calip GS, Agrawal V (2020). A multi-institutional comparison of mitoxantrone, etoposide, and cytarabine vs high-dose cytarabine and mitoxantrone therapy for patients with relapsed or refractory acute myeloid leukemia. Am J Hematol.

[119] Martinelli Boneschi F, Vacchi L, Rovaris M, Capra R, Comi G (2013). Mitoxantrone for multiple sclerosis. Cochrane Database Syst Rev.

[120] Martinelli Boneschi F, Rovaris M, Capra R, Comi G (2005). Mitoxantrone for multiple sclerosis. Cochrane Database Syst Rev.

[121] de Bono JS, Oudard S, Ozguroglu M, Hansen S, Machiels JP, Kocak I (2010). Prednisone plus cabazitaxel or mitoxantrone for metastatic castration-resistant prostate cancer progressing after docetaxel treatment: a randomised open-label trial. Lancet.

[122] Tannock IF, de Wit R, Berry WR, Horti J, Pluzanska A, Chi KN (2004). Docetaxel plus prednisone or mitoxantrone plus prednisone for advanced prostate cancer. N. Engl J Med.

[123] Jacko AM, Nan L, Li S, Tan J, Zhao J, Kass DJ (2016). De-ubiquitinating enzyme, USP11, promotes transforming growth factor β-1 signaling through stabilization of transforming growth factor β receptor II. Cell death Dis.

[124] Colland F, Formstecher E, Jacq X, Reverdy C, Planquette C, Conrath S (2009). Small-molecule inhibitor of USP7/HAUSP ubiquitin protease stabilizes and activates p53 in cells. Mol Cancer Therapeut.

[125] Reverdy C, Conrath S, Lopez R, Planquette C, Atmanene C, Collura V (2012). Discovery of specific inhibitors of human USP7/HAUSP deubiquitinating enzyme. Chem Biol.

[126] Tian X, Isamiddinova NS, Peroutka RJ, Goldenberg SJ, Mattern MR, Nicholson B (2011). Characterization of selective ubiquitin and ubiquitin-like protease inhibitors using a fluorescence-based multiplex assay format. Assay Drug Dev Technol.

